# Self-expanding vs. balloon-expandable transcatheter heart valves in small aortic annuli

**DOI:** 10.3389/fcvm.2023.1175246

**Published:** 2023-08-03

**Authors:** Anastasiya Kornyeva, Melchior Burri, Rüdiger Lange, Hendrik Ruge

**Affiliations:** ^1^ Department of Cardiovascular Surgery, German Heart Centre Munich at the Technical University Munich, Munich, Germany; ^2^ Insure (Institute for Translational Cardiac Surgery), Department of Cardiovascular Surgery, German Heart Center Munich at the Technical University of Munich, Munich, Germany; ^3^ DZHK (German Center for Cardiovascular Research)-Partner Site Munich Heart Alliance, Munich, Germany

**Keywords:** TAVR, SEV, BEV, small annuli, PPM

## Abstract

**Background:**

Clinical consequences of prosthesis–patient mismatch (PPM) after transcatheter aortic valve replacement (TAVR) is currently in the focus of clinical research. Patients with small aortic annulus are at higher risk to display PPM. Data on incidence and clinical consequences of PPM after TAVR with either balloon-expandable (BEV) or self-expanding (SEV) transcatheter heart valves in small aortic annulus are sparse.

**Methods:**

Patients with small aortic annulus (perimeter < 72 mm or aortic annulus area < 400 mm^2^) who underwent BEV or SEV with contemporary transcatheter heart valve types were identified from the institutional TAVR database. Propensity score matching was applied for imbalanced baseline characteristics between patients undergoing BEV or SEV. Echocardiography and clinical follow-up beyond 3 years was reported following VARC-3 recommendations. Primary endpoint was the incidence of pre-discharge PPM and its association with 3-year mortality.

**Results:**

From a total of 507 patients with small aortic annulus, 192 matched patient pairs with SEV or BEV were identified. Mean age was 81 ± 7 (SEV) vs. 81 ± 6 (BEV) years (*p* = 0.5), aortic annulus perimeter was 69 ± 3 vs.69 ± 3 mm, (*p* = 0.8), annulus area was 357 ± 27 vs.357 ± 27 mm^2^ (*p* = 0.8), and EuroScore II was 5.8 ± 6.6 vs.5.7 ± 7.2 (*p* = 0.9). SEV resulted in less moderate (20% vs. 31%, *p* < 0.001) and severe pre-discharge PPM (9% vs.18%, *p* < 0.001) compared to BEV. At discharge (7 ± 4 vs. 12 ± 9 mmHg, *p* = 0.003) and at 1-year follow-up (7 ± 5 vs.13 ± 3 mmHg, *p* < 0.001), SEV displayed lower mean gradients compared to BEV. Estimated survival after SEV was 85% (95% confidence interval (CI): 80%–90%) at 1 year, 80% (95% CI: 75%–86%) at 2 years, and 71% (95% CI: 65%–78%) at 3 years; estimated survival after BEV was 87% (95% CI: 82%–92%) at 1 year, 81% (95% CI: 75%–86%) at 2 years, and 72% (95% CI: 66%–79%) at 3 years, with no significant difference among the groups (*p* = 0.9) Body surface area (OR: 1.35, *p* < 0.001), implantation of BEV (odds ratio (OR): 3.32, *p* < 0.001), and the absence of postdilatation (OR: 2.16, *p* < 0.001) were independent risk factors for any PPM. At 3 years, patients without PPM had a higher 3-year survival compared with patients with ≥moderate PPM (77% vs. 67%, *p* = 0.03).

**Conclusion:**

BEV implantation in patients with small annulus was associated with a twofold higher incidence of pre-discharge severe PPM compared to SEV implantation. Survival at 3 years after TAVR was similar after BEV and SEV. However, patients with the absence of pre-discharge PPM had a higher 3-year survival compared to patients with ≥moderate PPM.

## Introduction

Transcatheter aortic valve replacement (TAVR) in patients with small aortic annuli may result in prosthesis–patient mismatch (PPM), whose consequences have been extensively investigated following the implantation of surgical aortic valves (SAVs). Only few data are available for its incidence after TAVR. PPM is defined as a condition where the orifice area of a prosthetic aortic valve is relatively too small related to the patient's body size (1). This condition often translates into increased transvalvular gradients (1). PPM is categorized to be moderate PPM with an indexed effective orifice area (iEOA) >0.65–0.85 cm^2^/m^2^ and severe PPM with an iEOA < 0.65 cm^2^/m^2^ (1). Following surgical aortic valve replacement (SAVR), moderate PPM is present between 20% and 70% and severe PPM between 2% and 20% (2). In a meta-analysis including >40,000 SAVR patients, PPM was associated with decreased short-term and long-term survival (3).

For TAVR, incidence and clinical significance of PPM is currently in the focus of intense research. Incidence of PPM is reported twofold higher after balloon-expandable (BEV) compared to self-expanding (SEV) TAVR (4). For patients with small aortic annulus, severe post-TAVR PPM is reported even sevenfold higher after BEV compared to TAVR with the Acurate Neo THV (5). Small aortic annulus and implantation of a BEV were previously identified as independent predictors for PPM (6). The present study aims to analyze the incidence of PPM after TAVR in patients with small aortic annulus using current generation SEVs and BEVs. Furthermore, a potential association between PPM and 3-year survival was analyzed and a risk factor analysis for post-TAVR PPM was performed.

## Materials and methods

Between September 2014 and June 2020, all out of 2,469 consecutive TAVR patients with small aortic annulus who underwent TAVR with a contemporary SEV and BEV were identified from our TAVR database. In our center, contemporary THV systems became available in September 2014. According to previously defined criteria, small aortic valve annulus was defined as CT-derived annular perimeter <72 mm or aortic annulus area <400 mm^2^ (7). Patients with a valve-in-valve procedure were excluded. Mean gradients and EOA were measured by transthoracic echocardiography (TTE) preoperatively and at discharge. Prosthesis–patient mismatch was categorized based on the iEOA: PPM was not present in patients with iEOA >0.85 cm^2^/m^2^, moderate for iEOA of 0.85–0.65 cm^2^/m^2^, and severe for iEOA <0.65 cm^2^/m^2^ (1, 2, 8).

### Endpoints

The primary endpoints were the incidence of post-TAVR PPM and its association with 3-year mortality.

Secondary endpoints included mean transvalvular gradient and paravalvular regurgitation at and 1 year after discharge. VARC-3-defined combined endpoints were reported: 30 days device success [technical success, freedom from mortality, freedom from reintervention related to the device, hemodynamic device performance (mean gradient <20 mmHg, peak velocity <3 m/s)] and modified 1-year clinical efficacy (freedom from all-cause mortality, freedom from stroke, freedom from procedure-related rehospitalization, and kansas city cardiomyopathy questionaire (KCCQ) score was not recorded) (9).

Major vascular complications were reported according to VARC-3 criteria at hospital discharge. Need for permanent pacemaker implantation and stroke (VARC-3 all stroke) were reported at 30 days (9).

### Data collection

Demographics, procedural details, intra-hospital course, and adverse events were prospectively recorded and reported according to the VARC-3 recommendations (9).

Echocardiography examination at discharge and at 1-year follow-up was performed with two-dimensional and Doppler TTE in accordance with the imaging recommendations of prosthetic heart valves (10). The EOA was measured at discharge by TTE using the continuity equation. The measurement was indexed for body surface area (BSA) to categorize PPM.

The Institutional Review Board of the Technical University of Munich approved the study (approved number of 540/20S).

### Statistical analysis

All statistical analyses were performed using R-statistical software (version 3.6.1, R Foundation for Statistical Computing, Vienna, Austria).

Categorical variables were presented as absolute numbers and percentages. A *χ*^2^ test (Fisher correction test) was used for categorical data, except for binomial variables with sufficiently low sample size, for which Fisher exact test was used. Continuous variables are expressed as mean ± SD or median with interquartile range (IQR), as appropriate.

An independent sample *t*-test was used to compare groups with normally distributed variables, and Mann–Whitney test was used for variables that were not normally distributed. Kaplan–Meier (KM) survival curve was computed to present all-cause mortality in patients with and without PPM, and in BEV and SEV groups, each endpoint was analyzed with KM. Differences in the endpoints were evaluated using the long-rank test. Propensity score matching was performed using non-parsimonious multivariate logistic regression. Because most variables were already balanced before matching, only the annulus perimeter, annulus area, and body surface area were entered into the logistic model to calculate the propensity score. The C-statistics of the logistic model was 0.60 and McFadden's pseudo R was 0.03 before matching. After matching, a corresponding model had a C-statistics of 0.51 and McFadden's pseudo R was 0.0004. The absolute standardized difference of means of the propensity score between the groups (Rubin's B) was 0.41 before and 0.02 after matching. The ratio of the variances of the propensity score between the groups (Rubin's R) was 0.67 before and 1.02 after matching.

Logistic regression was used to determine the risk factors for the development of an at least moderate PPM, and Cox regression was used to determine risk factors for death.

## Results

### Baseline characteristics

A total of 507 patients met the inclusion criteria with 310 patients undergoing TAVR with SEV [247 Evolut R/PRO (Medtronic, Minneapolis), 3 Portico (Boston Scientific, Marlborough), 60 Acurate Neo 2 (Abbott, Chicago)] and 197 individuals with BEV. Mean age was 81 ± 7 years, and the majority of patients were female (78%). Mean EuroScore II was 6% ± 7% and Society of Thoracic Surgeons predicted risk of mortality (STS-PROM) was 4.9% ± 4.8%. Baseline characteristics of the BEV and SEV groups are shown in Table 1.

**Table 1:** Baseline patient characteristics of the total and matched cohort.

Variables, mean ± SD or *n* (%)	Total cohort *N* = 507	BEV *N* = 197	SEV *N* = 310	*p*-value	Matched cohort *N* = 384	BEV *N* = 192	SEV *N* = 192	*p*-value
Female, *n* (%)	395 (78%)	156 (79%)	239 (77%)	0.3	294 (77)	153 (80)	141 (73%)	0.7
Age (years)	81 ± 7	81 ± 6	81 ± 7	0.4	81 ± 7	81 ± 6	81 ± 7	0.5
BSA (m^2^)	1.8 ± 0.2	1.8 ± 0.2	1.8 ± 0.2	0.7	1.8 ± 0.2	1.8 ± 0.2	1.8 ± 0.2	0.8
Annulus perimeter (mm)	68 ± 3	69 ± 3	67 ± 4	<0.001	69 ± 3	69 ± 3	69 ± 03	0.8
Annulus area (mm^2^)	351 ± 38	358 ± 27	344 ± 43	<0.001	357.6 ± 26.9	357.9 ± 27.1	357.3 ± 26.7	0.8
height (cm)	162 ± 10	163 ± 8	161 ± 12	0.08	162 ± 10	163 ± 8	161 ± 12	0.08
weight (kg)	70 ± 15	69 ± 15	70 ± 15	0.4	69 ± 15	69 ± 15	70 ± 16	0.7
Coronary artery disease	269 (53%)	106 (54%)	163 (53%)	0.9	215 (56%)	104 (54%)	111 (58%)	0.5
PAD	83 (16%)	40 (20%)	43 (14%)	0.06	68 (18%)	39 (20%)	29 (15%)	0.3
Carotid artery stenosis	50 (10%)	19 (10%)	31 (10%)	1.0	38 (10%)	19 (10%)	19 (10%)	1.0
Creatinine (mg/dl)	1.2 ± 0.6	1.2 ± 0.6	1.2 ± 0.5	0.3	1.1 ± 0.5	1.2 ± 0.6	1.2 ± 0.4	0.3
Urea (mg/dl)	49 ± 27	49 ± 27	49 ± 26	0.9	50 ± 28	50 ± 27	51 ± 29	0.7
BNP (ng/dl)	3,703 ± 5,975	3,773 ± 6,201	3,659 ± 5,841	0.9	3,913 ± 6,142	3,770 ± 6,240	4,058 ± 6,061	0.7
Preop pacemaker, *n* (%)	43 (8%)	16 (8%)	27 (9%)	0.9	35 (9%)	15 (8%)	20 (10%)	0.4
Atrial fibrillation, *n* (%)	121 (24%)	48 (24%)	73 (24%)	0.8	100 (26%)	48 (25%)	52 (27%)	0.7
Bicuspid valve, *n* (%)	23 (5%)	8 (4%)	15 (5%)	0.8	16 (4%)	8 (4%)	8 (4%)	1.0
NYHA, *n* (%)								
II	13 (3%)	5 (3%)	8 (3%)	0.7	7 (2%)	49 (26%)	61 (32%)	0.2
III	450 (89%)	173 (88%)	277 (89%)	0.7	339 (88%)	42 (22%)	43 (22%)	1.0
IV	38 (7%)	15 (8%)	23 (7%)	0.7	34 (9%)	17 (9%)	20 (10%	0.7
Porcelain aorta	48 (9%)	17 (9%)	31 (10%)	0.6	37 (10%)	17 (9%)	20 (10%)	0.7
EuroScore II	5.5 ± 6.5	5.7 ± 7	5.4 ± 6.1	0.6	5.8 ± 6.9	5.7 ± 7.2	5.8 ± 6.6	0.9
STS-PROM	4.9 ± 4.8	4.8 ± 4.7	5.0 ± 4.8	0.7	4.9 ± 4.8	4.8 ± 4.9	5.0 ± 4.8	0.7

PAD, peripheral artery disease; BNP, brain natriuretic peptide; NYHA, New York Heart Association.

Differences among the BEV and SEV groups were found in CT-derived aortic annulus perimeter (69 ± 3 vs. 67 ± 4 mm, *p* < 0.0001) and CT-derived aortic annulus area (358 ± 27 vs. 344 ± 43 mm^2^, *p* < 0.0001).

Propensity score matching for imbalanced baseline CT-derived aortic annulus perimeter and area resulted in 192 patient pairs (Table 1). Within the SEV group, 148 were Evolut R/PRO, 42 Acurate neo 2, and 2 Portico THV.

### Incidence of PPM and impact on survival

**Figure 1:**
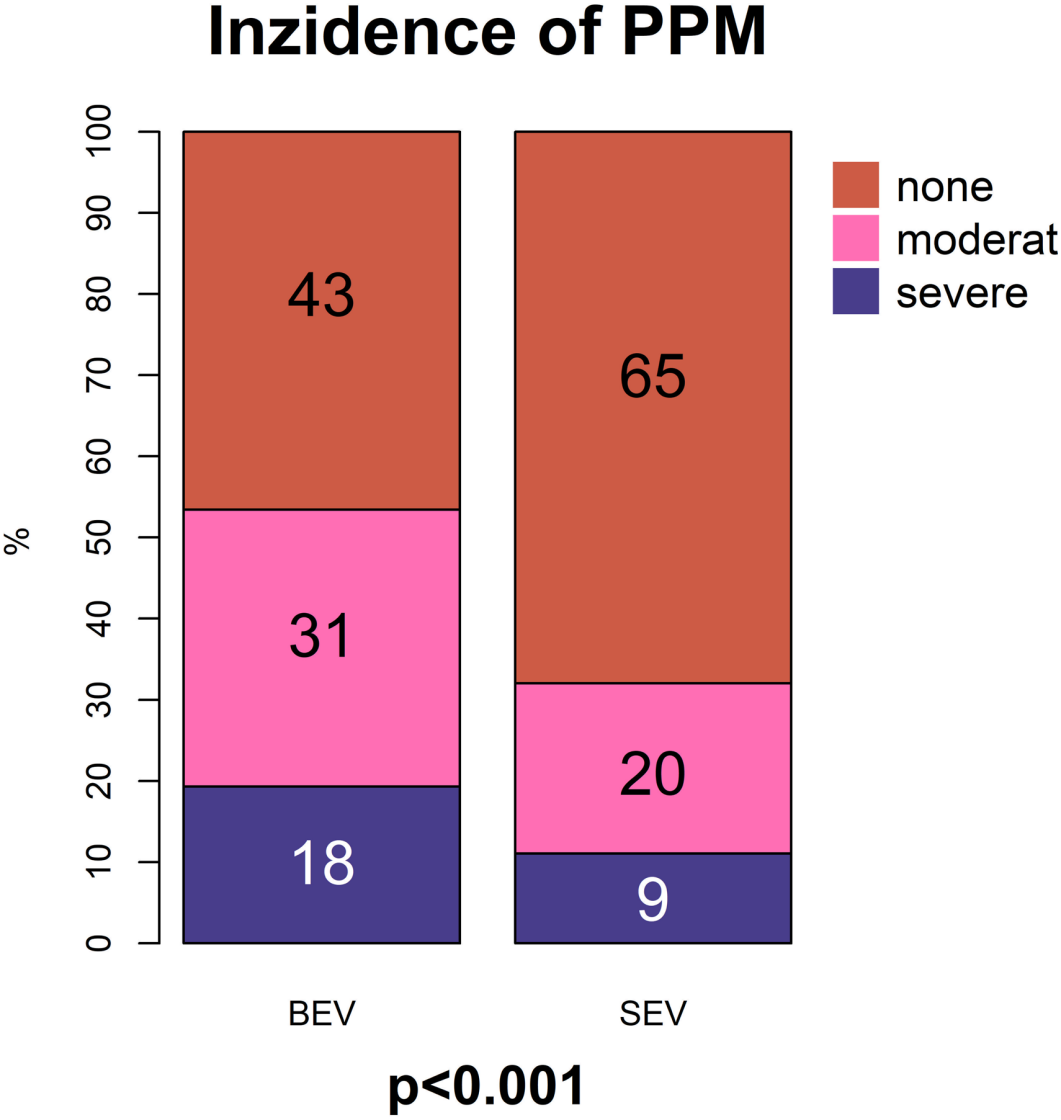
Diagram of incidence of the PPM after TAVR with SEV und BEV.

About 151 (39%) patients showed PPM, 99 (26%) moderate PPM and 52 (14%) severe PPM. Rate of moderate (31% vs. 20%, *p* < 0.001) and severe PPM (18% vs. 9%, *p* < 0.001) was higher in BEV compared to SEV (Figure 1).

**Figure 2:**
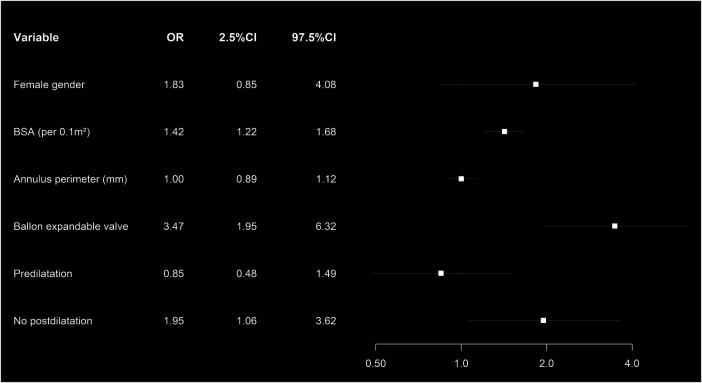
Forest plot showing the multivariate analysis of the risk factors associated with moderate and severe PPM in matched patients’ cohort.

**Figure 3:**
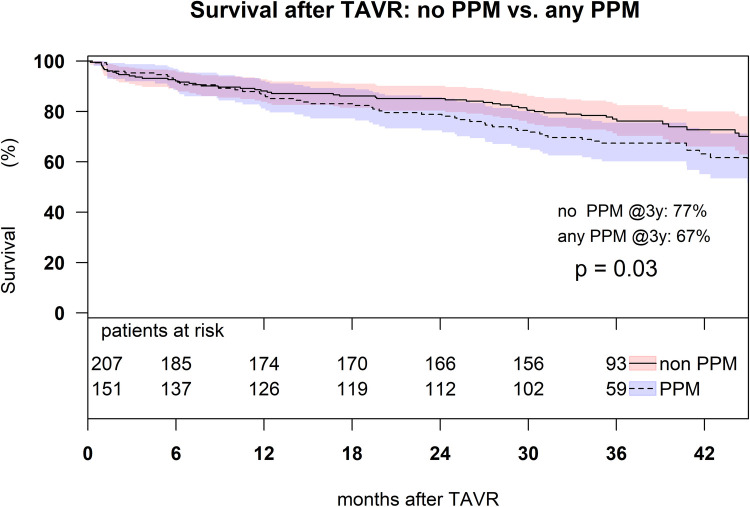
Kaplan–Meier survival analysis up to 3 years in the matched patient cohort with moderate and severe PPM comparing to the patients without PPM (*p* = 0.03).

Independent risk factors associated with moderate and severe PPM were BSA (odds ratio (OR): 1.35, *p* < 0.001) implantation of a BEV (OR: 3.32, *p* < 0.001), and the absence of postdilatation (OR: 2.16, *p* < 0.013) (Figure 2). Patients without PPM had a higher 3-year survival (76%, CI: 0.66–0.79) compared to patients with ≥moderate pre-discharge PPM (67%, CI: 0.60–0.76, *p* = 0.03) (Figure 3).

### Valve performance

**Figure 4:**
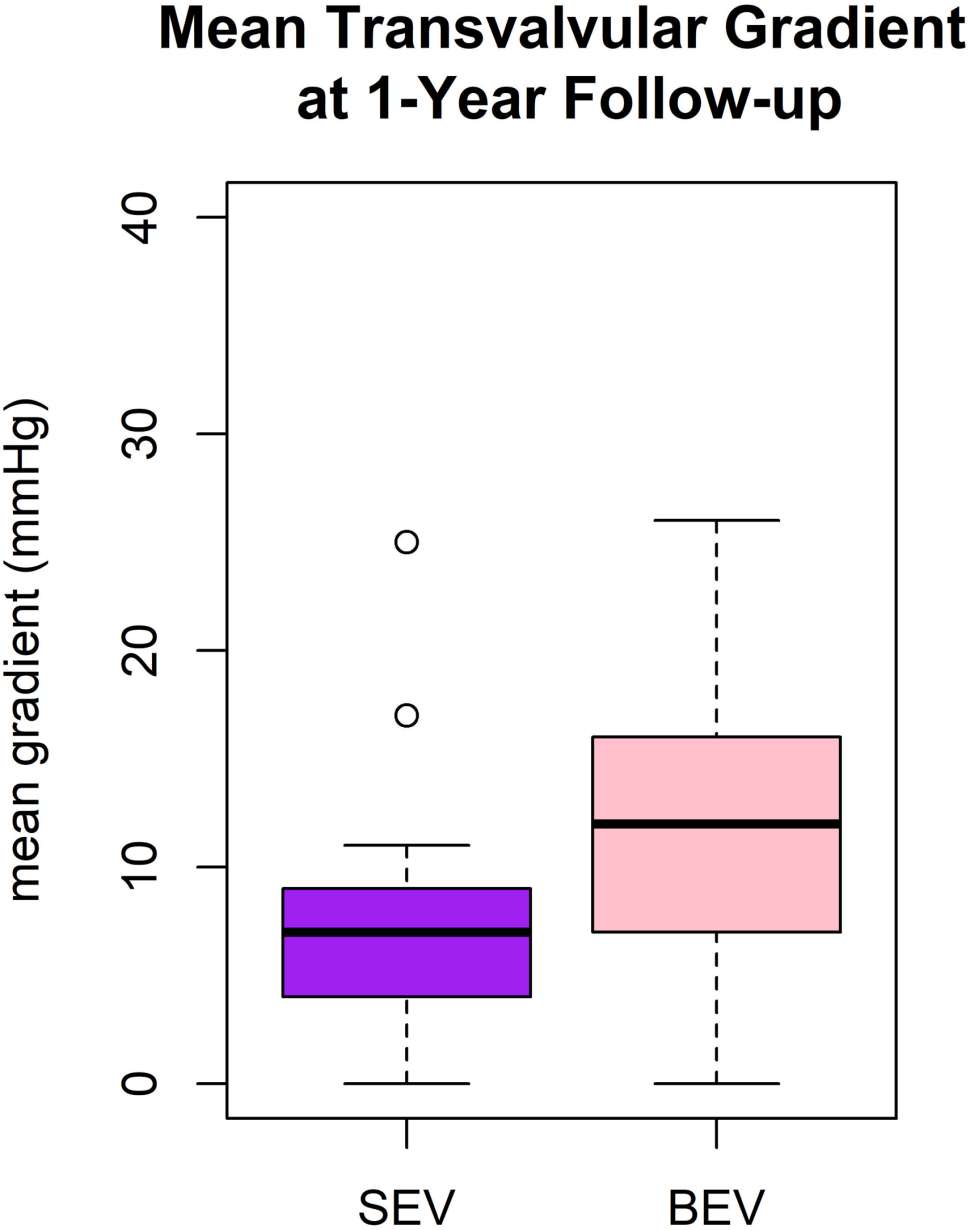
Boxplot of mean transprosthetic gradient at 1-year follow-up after TAVR with SEV and BEV.

Mean transvalvular gradient was reduced from 40 ± 16 to 10 ± 8 mmHg (*p* < 0.0001). At discharge, mean transvalvular gradient was 12 ± 9 mmHg in the BEV group and 7 ± 4 mmHg in the SEV group (*p* = 0.003). At 1-year follow-up, mean transvalvular gradient was 13 ± 3 mmHg in the BEV group and 7 ± 5 mmHg in the SEV group (*p* < 0.001) (Figure 4).

Echocardiographic findings of paravalvular leak (PVL) at and at 1 year after discharge are displayed in Table 2. VARC-3 device success [technical success, freedom from mortality, freedom from reintervention related to the device, and hemodynamic device performance (mean gradient <20 mmHg, peak velocity <3 m/s)] at 30 days was 86% in SEV and 84% in BEV (*p* = 0.8). VARC-3 clinical efficacy (freedom from all-cause mortality, freedom from stroke, freedom from procedure-related rehospitalization, KCCQ score was not recorded) at 1 year was 79% in BEV and 80% in SEV (*p* = 0.3).

**Table 2:** Valve performance.

Variables	BEV *N* = 192	SEV *N* = 192	*p*-value
Preoperative mean AV gradient, ±SD	41 ± 16	39 ± 16	0.1
Mean transprosthetic gradient at discharge, ±SD	12 ± 9	7 ± 4	<0.001
Mean transprosthetic gradient at 1-year follow-up, ±SD	13 ± 13	7 ± 5	0.002
PVL at discharge			
None/trace	80%	77%	0.4
mild	19%	23%	0.3
PVL ≥ moderate	1%	0%	1
PVL at 1 year			
None/trace	92%	86%	0.1
mild	7%	17%	0.003
PVL > moderate	1%	1%	1

AV, aortic valve.

### Survival

**Figure 5:**
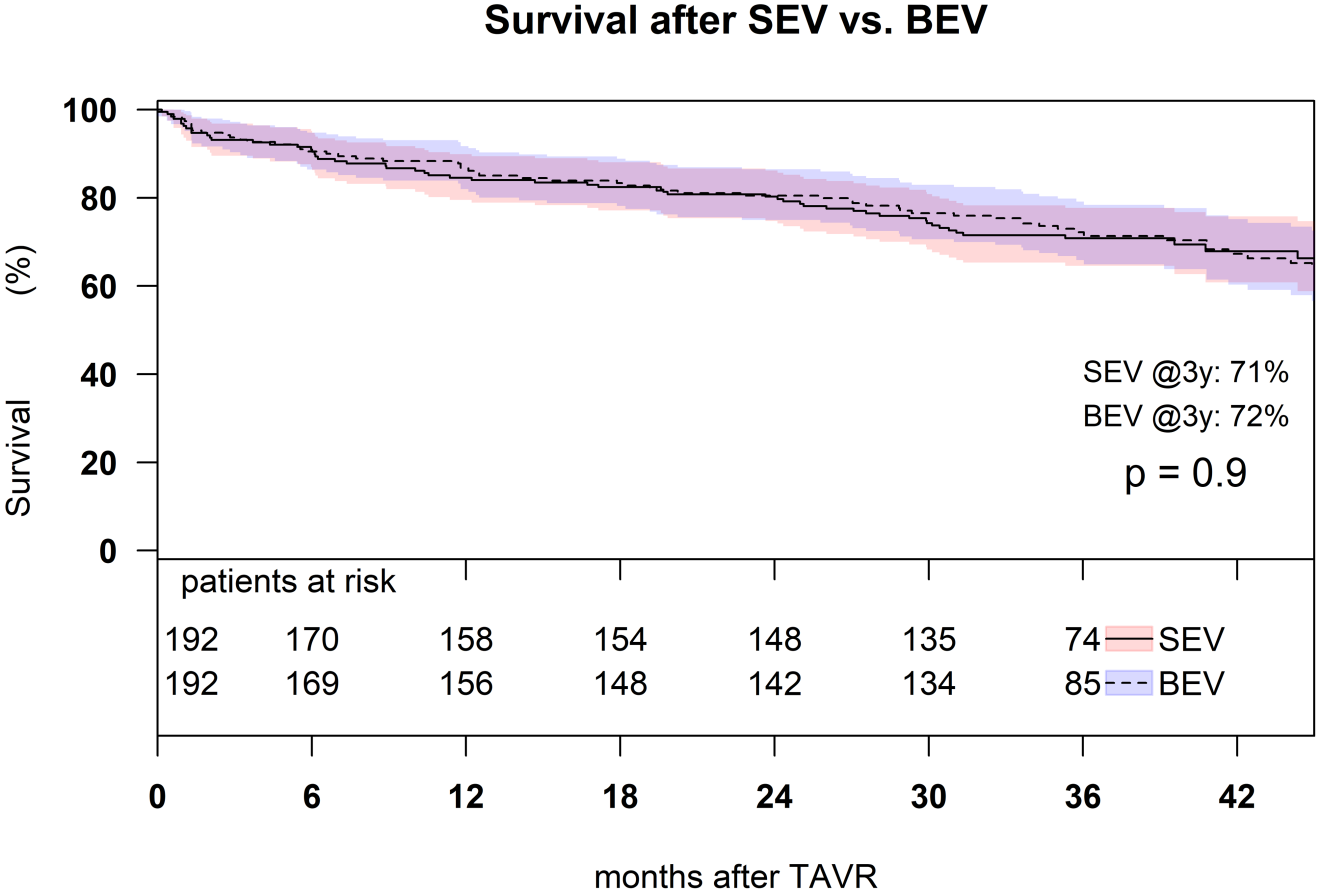
Kaplan–Meier survival analysis of 3-year survival after TAVR with SEV and BEV in the matched cohort (*p* = 0.9).

Estimated survival after SEV was 85% (95% confidence interval (CI): 80%–90%) at 1 year, 80% (95% CI: 75%–86%) at 2 years, and 71% (95% CI: 65%–78%) at 3 years; estimated survival after BEV was 87% (95% CI: 82%–92%) at 1 year, 81% (95% CI: 75%–86%) at 2 years, and 72% (95% CI: 66%–79%) at 3 years, with no significant difference among the groups (*p* = 0.9) (Figure 5).

**Figure 6:**
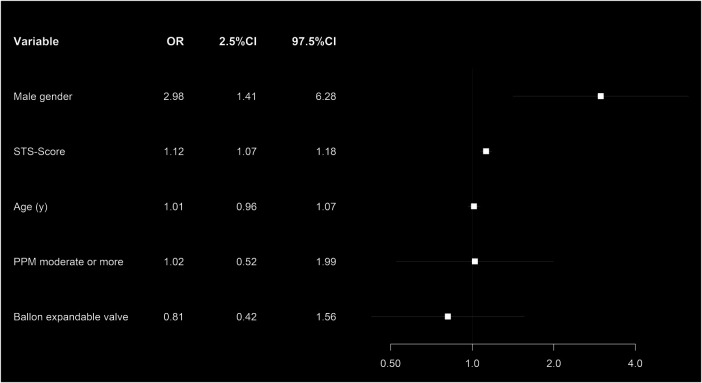
Forest plot showing the multivariate risk factor analysis associated with 1-year all-cause mortality in the propensity score matched cohort.

Male gender (OR: 2.25, *p* = 0.045) and increasing STS-PROM (OR: 1.08, *p* < 0.001) were associated with all-cause mortality after BEV and SEV (Figure 6).

### Vascular complications, stroke, and permanent pacemaker implantation after TAVR

At discharge, VARC-3 major vascular complications occurred in 13% of patients after BEV and in 9% of patients after SEV (*p* = 0.3). At 30 days, permanent pacemaker implantation was required in 5% with BEV and 6% with SEV (*p* = 0.6) (Table 3).

**Table 3:** 30-day vascular complications, stroke, and pacemaker.

Variables	Matched patients *N* = 384	BEV *N* = 192	SEV *N* = 192	*p*-value
Major vascular events	43 (11%)	25 (13%)	18 (9%)	0.3
Pacemaker	22 (6%)	10 (5%)	12 (6%)	0.6
Non-disabling and disabling stroke	23 (6%)	13 (7%)	10 (5%)	0.7

At 30 days, rate of stroke was 7% with BEV and in 5% with SEV (*p* = 0.7) Table 3.

## Discussion

To our knowledge, the present study reports on the largest, so far published patient cohort, comparing SEV and BEV in patients with small aortic annulus using propensity score matching for imbalanced baseline aortic annulus dimensions and follow-up beyond 3 years.

The main findings of the study are as follows:

• Incidence of moderate and severe pre-discharge PPM was significantly higher after BEV compared to SEV.
• Transvalvular gradients were significantly higher at 30-days and 1-year after BEV compared to SEV.
• All-cause 3-year mortality did not differ after SEV and BEV.
• PPM was associated with 3-year mortality.

### Transvalvular gradients, prosthesis–patient mismatch, and survival

In the present study, single digit mean transvalvular gradients were achieved in patients with small aortic annulus after SEV using predominantly the Medtronic Evolut R and PRO prostheses. The TAVI-SMALL registry analyzed 859 SEV patients with small aortic annulus applying the same sizing criteria as in our study. Comparable low transvalvular gradients were reported using SEVs, i.e., Medtronic’s Evolut R and PRO prostheses, Boston Scientific’s Acurate Neo, and Abbott St. Jude’s Portico (11). Compared to SEV, we found higher transvalvular gradients after BEV at and 1 year after discharge (*p* < 0.001). Mauri compared the Acurate Neo and the Sapien 3 in 92 propensity matched patient pairs with small annulus applying equal annulus size criteria as in our study and the TAVI-SMALL registry (6). SEV likewise resulted in lower transvalvular gradients at 30 days and 1 year compared to SEV (6). The TAVI-SMALL 2 registry analyzed 1,378 patients with small annulus. In line with our findings, higher transvalvular gradients were reported after BEV compared to SEV (12).

In our study, the lower transvalvular gradients after SEV translated into lower incidence of severe PPM compared to BEV (9% vs. 18%, *p* < 0.001). In the TAVI-SMALL registry, for SEV, a similar rate of 9.4% severe PPM is reported (11). Comparing SEV to BEV, Mauri reported a sevenfold higher incidence of severe PPM after BEV (3% vs. 22%, *p* = 0.004) in a propensity matched cohort (6). TAVI-SMALL 2 with imbalanced aortic annulus dimensions reports a more than twofold higher incidence of severe PPM with BEV (5.6% vs. 14.2%, *p* < 0.001) (12).

PPM in patients with small aortic annulus becomes clinically significant beyond 2 years after TAVR. At 1-year follow-up, we found no association of severe PPM to mortality neither after SEV or BEV. Similarly, (6) reported no difference in 1-year mortality after SEV and BEV (SEV 8.3% vs. BEV 13.3%, *p* = 0.233). Longer surveillance reveals an association of post-TAVR PPM with mortality. We found higher 3-year mortality rates in patients with any pre-discharge PPM. Likewise, the TAVI-SMALL registry reported severe PPM to be independently associated with 2-year mortality (12). In a study of 1,309 all-comer TAVR patients, severe PPM was an independent risk factor for 3-year mortality for a subgroup of patients with an ejection fraction <40% (13). Leon del Pino reported 6% severe PPM in a single-center study including 185 patients (14). Severe PPM was associated with a lower event-free survival (death, stroke, or hospitalization for heart failure) at 34 months (52% vs. 84%) (14). A meta-analysis including almost 82,000 patients showed an association between severe PPM and mortality after TAVR (15).

We found BSA, TAVR without postdilatation, and implantation of a BEV as risk factors for post-TAVR PPM. Likewise, the OCEAN registry including Japanese patients only identified implantation of a BEV, younger age, and small aortic annulus as risk factors for post-TAVR PPM (7). In the TAVI-SMALL registry, use of an intra-annular SEV was identified as a risk factor for post-TAVR PPM, while THV-oversizing and intraprocedural postdilatation reduced the incidence of severe PPM (11).

### Future perspectives

With growing evidence of the association of PMM with impaired midterm survival after TAVR in small aortaic annulus, prospective data are required. The prospective and randomized SMART trial (ClinicalTrials.gov Identifier: NCT04722250) completed enrollment in September 2022 and compares TAVR patients with small aortic annulus receiving either the Medtronic Evolut PRO or Pro+ or the Edwards Sapien 3/3 ultra. With a 5-year follow-up, the SMART trial will analyze clinical outcomes (mortality, stroke, and heart failure rehospitalization) and valve function (PPM and PVL among other endpoints).

### Study limitations

In this study, we used the data from a single-center registry. The study is limited by its retrospective and non-randomized design. Selection and confounding bias cannot be excluded. Follow-up transthoracic echocardiography was performed by multiple echocardiographers. No core lab evaluation was performed for baseline computerized tomography and baseline and follow-up echocardiography.

## Conclusion

BEV implantation in patients with small annulus was associated with a twofold higher incidence of pre-discharge severe PPM compared to SEV implantation. Survival at 3 years after TAVR was similar after BEV and SEV. However, patients with the absence of pre-discharge PPM had a higher 3-year survival compared to patients with ≥moderate PPM.

## Data Availability

The original contributions presented in the study are included in the article, further inquiries can be directed to the corresponding author.
